# Assessment of the Quality of Life of Women after Osteoporotic Vertebral Fracture with Consideration of Socio-Demographic Characteristics and Selected Factors Concerning the State of Health

**DOI:** 10.3390/ijerph191912237

**Published:** 2022-09-27

**Authors:** Małgorzata Dziedzic, Mariola Janiszewska, Małgorzata Goździewska, Wioleta Kowalska, Jacek Roliński

**Affiliations:** 1 Independent Public Regional Specialist Hospital in Chełm, Ceramiczna 1, 22-100 Chełm, Poland; 2 Department of Medical Informatics and Statistics with e-Health Lab, Medical University of Lublin, K. Jaczewskiego 5 Street, 20-059 Lublin, Poland; 3 Department of Medical Anthropology, Institute of Rural Health, 20-090 Lublin, Poland; 4 Department of Clinical Immunology, Medical University of Lublin, 20-093 Lublin, Poland

**Keywords:** osteoporosis, osteoporotic vertebral fracture, quality of life, self-estimation of state of health, life style

## Abstract

**Introduction**: Fractures of the vertebral bodies are a frequent complication of osteoporosis, hospitalization, decline in physical fitness and, in consequence, deterioration in the quality of life. **Objective**: The aim of the study was assessment of the quality of life according to the QUALEFFO-41 questionnaire in patients who had undergone fractures of the vertebral bodies, and presentation of the relationships between the quality of life, socio-demographic characteristics, and selected factors concerning the state of health. **Materials and Method**: The study included 243 women with osteoporotic vertebral fractures, and was conducted in the Outpatient Departments for the Treatment of Osteoporosis in the city of Lublin (eastern Poland). For the purposes of the study, the Quality of Life Questionnaire (QUALEFFO-41) and the author’s questionnaire were employed, and Spearman’s rank correlation coefficient, t-Student test, and Tukey test were used, along with analysis of variance (ANOVA). The level of statistical significance was set at α = 0.05. **Results**: The quality of life of woman with vertebral compression fractures remains on a mediocre level. Significant relationships were observed between the respondents’ quality of life and certain socio-demographic characteristics, duration of the disease, and complaints related with osteoporosis. **Conclusions**: It is important to implement appropriate therapy and provide comprehensive, holistic care to women after fractures.

## 1. Introduction

Among the main problems for women at post-menopausal age are increasing functional disorders that undeniably affect their quality of life. The loss of bone mineral density which increases with age and is related to the ageing of the skeletal system begins in women from the age of about 40. Decrease in the production of estrogens occurring during menopause results in the rapid loss of bone mass. One of the chronic diseases affecting older women is osteoporosis, the consequence of which are skeletal system disorders and reduced bone strength which increase the risk of brittle fractures [1,2]. These bone fractures are the most important clinical symptoms of osteoporosis [3,4]. Osteoporosis is a disease with a slow, latent course, and frequently is not manifested with any preceding symptoms prior to the occurrence of the fracture, while these fractures and their consequences are most important from the clinical aspect [5]. According to the International Osteoporosis Foundation (IOF) and the European Federation of Pharmaceutical Industry Association (EFPIA), 22 million women and 5.5 million men were estimated to have osteoporosis in 27 European countries [6]. In Poland, approximately 30–40% women at postmenopausal age are challenged by osteoporosis [7]. Kanis et al. anticipate that due to demographic changes in the population worldwide, the annual number of fractures in persons with osteoporosis will increase from 3.5 million in 2010 to 4.5 million in 2025 [8,9]

Disturbances of consciousness, vision and hearing, as well as past diseases of the nervous or cardiovascular systems, are also frequently observed [10]. The result of an osteoporotic fracture is a considerable decrease in the quality of life. In the case of an osteoporotic vertebral fracture, the factors of greatest importance are pain complaints, limitation of the capability for self-care and physical fitness [11], as well as the limitation of social functions classified into the markers of the quality of life [12]. Cooper et al. [13] reported that during the first three months after low-energy fracture, due to persistent pain, reduced mobility, and dependence on others, there occurs a considerable decrease in the quality of life, irrespective of the location of the fracture [13].The study International Costs and Utilities Related to Osteoporotic Fractures Study (ICUROS) showed that the quality of life considerably deteriorates directly after bone fracture, and only after about 18 months after the fracture is an improvement observed in the psychological state, although it still remains lower than before the fracture [14].

Vertebral fractures are the most frequent type of osteoporotic fractures and occur in approximately 30–50% patients with osteoporosis [15]. During the first year after symptomatic vertebral fracture, patients experience a progressive decline in the quality of life, which continues to be felt even five years after the injury, most often related to chronic pain syndrome and the necessity to use analgesics [11,13]. Unfortunately, these fractures often remain undiagnosed, despite their relation with a considerable morbidity, and even mortality [16]. As many as 70% of fractures concerning the spine are not manifested by any symptoms. A past fracture of the vertebrae means up to a 3-fold increase in the risk of occurrence of subsequent osteoporotic fractures, and as high as an 11-fold increase in the risk of a subsequent fracture of the spine. [16]. Fractures of the vertebral bodies are the problem in every fourth woman aged over 50, and every second woman aged over 85, and both are burdened with mortality on the level of 15–20% within the period of five years after fracture [17]. Despite the fact that vertebral fractures are not always accompanied by noxious pain complaints, the loss of functionality and deterioration of the quality of life progresses with the number of fractures [13,18].

In the light of the ageing of society, and a constant increase in the number of the ill and disabled, it is important not only to extend life by treatment, but also to enable such ill or cured patients to lead a life as close as possible to that of a healthy one. For an ill person, the measure of effective treatment is primarily an improvement in wellbeing and overall fitness and functionality [19]. Low-energy osteoporotic fractures are most often sustained by the elderly, which is frequently associated with the burden of other chronic diseases; therefore, it is advisable to adopt proper strategy of treatment, making a reasonable choice between conservative and surgical treatment. An indispensable element of treatment after compressive fracture of the vertebrae is application of a long-term pharmacological therapy of osteoporosis which, when implemented appropriately early offers a chance of avoiding subsequent fractures [8,17,18]. Among drugs used in pharmacotherapy after past fractures the following are worth mentioning: aminobisphosphonates—the effect of the new generation of bisphosphonates is based on the inhibition of bone tissue resorption, with the elimination of the unfavourable phenomenon of bone mineralization, which takes place in the case of simple bisphosphonates. Bisphosphonates belong to the group of drugs of a high anti-fracture effectiveness concerning all locations of fractures, and currently are fairly often applied in the treatment of osteoporosis [17]. Alternatives include the *selective oestrogen receptor modulators*, SERM are a non-steroid drug binding to oestrogen receptors, designed for postmenopausal women with low bone mineral density, both after and without fractures. Another drug is denosumab, which belongs to the group of drugs suppressing bone resorption by inhibiting osteoclastogenesis and activity of osteoclasts. A further option is teriparatide, a drug of high effectiveness designed for treatment of advanced primary and corticosteroid-induced osteoporosis, offered to patients with fractures and low level of BMD, frequently after recognition of ineffectiveness of therapy with antiresorptive drugs [17,18]. The next most common drug is strontium ranelate which both has antiresorptive action and stimulates the building of new bone tissue by suppressing the activity of osteoblasts and stimulation of pro-osteoblasts. Salmon calcitonin is also administered in the treatment. This is a polypeptide hormone secreted by thyroid C cells, the action of which is based on the inhibition of the activity of osteoclasts. Among new drugs which may be applied in the treatment of osteoporosis and fractures worth mentioning are: romosozumab which has a blocking effect on sclerostin, and is an osteocyte-derived protein inhibiting the process of bone formation; and abaloparatide, a parathormone [8,17,18,19].

It should be remembered that for an ill person, the measure of the effectiveness of treatment is primarily an improvement in efficiency within the range of daily activities, in wellbeing, and in satisfaction with life. Osteoporosis is also a predominantly female pathology: among diseases afflicting women more than men, osteoporosis is in the first place [20,21]. Therefore, the current study concerns women as well as the problems of respondents burdened with osteoporotic fractures, and is important from the scientific point of view.

Considering the scarce amount of research concerning the presented problem, the main aim of the study was a general assessment of the quality of life of women who had undergone osteoporotic vertebral fracture, according to their socio-demographic situation and selected factors pertaining to their state of health.

## 2. Materials and Methods

### 2.1. Study Design

The study design was cross-sectional and descriptive as part of a bigger project: ‘Acceptance of disease and the quality of life of women after osteoporotic vertebral fracture.’ The study was conducted in the Outpatient Department for the Treatment of Osteoporosis in the city of Lublin, and in the Lublin Diagnostic Centre in Świdnik, both in eastern Poland, during the period January 2016–April 2017.

Selection of respondents was random and purposive. The indispensable element of randomness was introduced by inviting for the study every second available woman. All the qualified patients who visited health facilities for medical advice were sequentially enrolled into the study. The women were voluntarily recruited during visits to outpatient clinics where the consent for the study was obtained. Approximately 70% of compression fractures of the vertebrae take a nearly asymptomatic course resulting in limited possibilities of selection of women who satisfied the criteria of inclusion into the study; therefore, the research material was collected very carefully and with utmost diligence. In all women included into the study, the fact was confirmed of past low energy fracture of the vertebral bodies in the course of osteoporosis, based on the analysis of medical records (radiographic examination of the spine, morphometric densitometry, computed tomography, or magnetic resonance). Also, in all respondents, the bone mineral density index T-score was analyzed, having been assessed in the lumbar vertebrae L1 to L4 and/or located anywhere around the proximal end of the femur. The study was voluntary, and each participant signed a consent form and was assured that the study was anonymous. The respondents were informed about the purpose of the study and about the method of completing the questionnaire. One of the researchers was on hand to help in the case of any difficulties in terms of understanding the language in the questionnaire. The majority of the questionnaires were completed independently or with little help, and the questions were defined as understandable and not difficult.

### 2.2. Participants

Due to the lack of data concerning the number of women who had undergone osteoporotic spine fracture in the Lublin Province of eastern Poland, the calculations regarding the size of the sample were based on estimations, taking into account the following data:

- life risk of spine fracture in women aged over 50 is 16% [16];
- the number of women aged 50–80 in the Lublin Province is 378,000 [22].

There is no reason to assume that individual provinces differ significantly according to the susceptibility of women to osteoporotic fractures. Therefore, it may be estimated that in the Lublin Province, approximately 60,500 women aged 50–80 sustained spine fracture with a background of osteoporosis, which is the size of the studied population. Based on the estimations, proper calculations were performed associated with the selection of the sample.

The study included 243 women. Assuming a confidence interval of 95%, the maximum error was 6%. This means that with only 5% risk of error, it may be estimated that a given result in the examined population may reflect a figure 6% higher or lower than for the total population. The inclusion criteria were as follows:

- women who had undergone osteoporotic vertebral fracture;
- women aged 50–80;
- post-menopausal women with natural cessation of the ovarian function, and women after premature menopause (cessation of the ovarian function before the age of 40, spontaneous or after resection of the ovaries, or chemo- or radiotherapy);
- women who were patients in the Outpatient Department for the Treatment of Osteoporosis located in the city of Lublin, and in the Lublin Diagnostic Centre in Świdnik, eastern Poland;
- written consent to participate in the study and understanding of the essence of the research.

### 2.3. Questionnaires

The authors applied two research instruments. The quality of life was measured using the Quality of Life Questionnaire of the European Foundation for Osteoporosis (QUALEFFO-41). The QUALEFFO-41 questionnaire is recommended by the International Osteoporosis Foundation (IOF) for investigating the quality of life of women at post-menopausal age after vertebral fractures. The questionnaire contains 41 items assessing five domains of functioning:

- pain (back pain, sleep disorders), items 1–5;
- physical functioning (physical daily activities, housework, mobility, appearance), items 6–22;
- social functioning (leisure time, sports, social activity, hobby), items 23–29;
- overall perception of health (changes in the quality of life), items 30–32;
- psychological functioning (mental performance, energy, hope, depression, fatigue, loneliness, anxiety), items 33–41.

The range of scores on the questionnaire is 0–100 points. The higher the mean score, the lower the quality of life [23]. Validation in the Polish version of the scale and assessment of psychometric properties was carried out under the supervision of Bączyk [24] and Lips [25] by a group of experts in the field of osteoporosis at the European Foundation for Osteoporosis. The reliability of the QUALEFFO-41 scale as assessed by the internal consistency coefficient (Cronbach’s α) is relatively high. The Cronbach’s α internal consistency coefficient is 0.72 for the entire scale, while for individual subscales, the values are 0.72 for general perception of health, and 0.77–0.92 for physical functioning [23].

Analysis of results of QUALEFFO-41 was performed according to the algorithm proposed by the International Osteoporosis Foundation. While calculating the mean and median values for all respondents, the level of the quality of life was assessed in each domain of the quality of life according to the QUALEFFO-41 questionnaire, and relatively diverse results were obtained.

The second research tool was an author-constructed questionnaire (Supplementary File S1: Research Questionnaire). It was designed according to the survey method for analyses of the relationships between variables and allowed the identification of the cause–effect relationships between them. The Questionnaire concerns socio-demographic data (age, place of residence, level of education, marital status, occupational activity); information concerning past vertebral fractures; and complaints associated with them and frequency of occurrence, assessed on the basis of respondents’ subjective evaluations. Apart from this, the questions concerned the method of diagnostics and treatment. The research tool also allows the supplementation of knowledge concerning self-reported health, prophylaxis of osteoporosis and fractures, and the respondents’ quality of life. After inducting the pilot study, the author-constructed Questionnaire was modified and evaluated in terms of content and form, and performed by the external company Market Research World (MRW), Gliwice, Poland.

### 2.4. Ethical Issues

The Bioethics Committee at the Medical University in Lublin approved the study in accordance with the requirements of the Helsinki Declaration (Decision No. KE 0254/76/2016). Each of the survey participants was informed of the purpose of the study, its voluntary nature and anonymity. All the respondents who met the criteria for inclusion in the study provided their consent, which they confirmed in writing. To ensure anonymity, the subjects were assigned encrypted codes. The researcher did not remain in any relationship of dependence with the respondents. Respondents were also informed of the possibility of withdrawal at any time during the data collection process.

### 2.5. Statistical Analysis

In order to present the results of our own research, basic descriptive statistics were used such as: arithmetic mean (M), standard deviation (SD), median (Me), minimum (Min.) and maximum (Max). Before parametric tests were applied, assumptions on the normality of distribution were verified by means of the Shapiro–Wilk test. Student’s t-test was used to examine whether there were any statistical differences in terms of the ratio variables between the two groups. Pearson’s correlation coefficient (r) was applied to investigate whether two quantitative variables were related by a linear relationship for variables from normal distribution. In addition, Spearman’s rank correlation was applied when one or both of the variables did not present characteristics of normal distribution or ratio scale. Analysis of variance (ANOVA) was applied to identify if there was a statistically significant difference concerning ratio variables between more than two groups. In order to examine which groups significantly differed from each other, a Turkey’s multiple comparison test was performed. A *p*-value of <0.05 defined the statistical significance of differences. Analyses were performed using Statistica software (Kraków, Poland).

## 3. Results

The study included 243 women who had undergone fractures of vertebral body due to osteoporosis. Their mean age was 68.97 ± 7.61 years old; respondents’ mean body weight was 63.84 ± 11.28 kg, mean height was 156.22 ± 7.37 cm; and Body Mass Index (BMI) for the whole examined group was 26.21 ± 4.67 kg/m^2^. The greatest majority of respondents were married or in a partnership relationship (58%; *n* = 141); 57.6% were urban inhabitants (*n* = 140), while 42.4% were rural inhabitants (*n* = 103). A similar number of respondents had primary vocational education (30.9%; *n* = 75) and secondary school education (29.6%; *n* = 72), and only 12.8% of respondents had higher education (*n* = 31). More than a half of respondents (59.7%; *n* = 145) evaluated their life conditions as sufficient. As many as 76.1% of respondents (*n* = 185) lived on retirement pension or disability allowance 76.1% (*n* = 185), whereas only 18.1% (*n* = 44) remained occupationally active. The greatest number of respondents (*n* = 98; 40.3%) had received treatment for osteoporosis for 1–5 years. In turn, every second woman in the study (*n* = 65; 26.7%) had received treatment for 5–10 years, and every fifth (*n* = 48; 19.8%) for longer than 10 years. Only 13.2% of respondents had been treated for less than one year (*n* = 32). All women sustained bone fractures, and had densitometry performed; in addition, 98.4% (*n* = 239) had radiological examination of the spine performed, every second—magnetic resonance 26.3% (*n* = 64), every fifth—computed tomography (21%; *n* = 51), and 73.4% (*n* = 179)—laboratory tests of blood and urine; however, in only 11.9% (*n* = 29) had the risk of bone fracture been assessed earlier using the fracture-risk algorithm-FRAX.

The respondents were asked about complaints related with past osteoporotic vertebral fracture, and highly frequently (*n* = 225; 93%) indicated chronic back pain at rest and/or while performing activities of daily life. More than a half of the women in the study (*n* = 140; 57%) experienced a decrease in height, nearly a half experienced acute pain in the mid–lower area of the thoracic spine or lumbar–thoracic spine (*n* = 107; 44.2%), and reduced mobility in the joints (*n* = 105; 43.4%). In turn, two in five women indicated difficulties with sitting down or standing up (*n* = 98; 40.5%), and rounding of the back or slouching (*n* = 92; 38.5%). Every third respondent (*n* = 79; 32.5%) had problems with locomotion, and 28.5% of the total number of respondents complained of disturbed bowel function (*n* = 69). Every fourth woman mentioned deterioration of mood, depression (*n* = 63; 26%), and a decrease in cardiovascular and respiratory efficiency (*n* = 60; 24.8%), and nearly every fifth woman (*n* = 44; 18.2%) experienced intensification of pain while sneezing, coughing and defecation. Only 1.7% of respondents did not experience any symptoms.

Nearly a half of respondents 49% (*n* = 119) had experienced pain in the area of the spine and back for more than 10 years. Frequent pain complaints in the area of the spine and back were reported by 64.2% of respondents (*n* = 156), while constant pain was experienced by 12.3% of respondents (*n* = 30). Prior to examination, more than a half of respondents were unaware of sustaining osteoporotic vertebral fracture. Two in three respondents (65%; *n* = 158) sustained fracture in the lumbar region of the spine. In turn, three in four women (74.5%; *n* = 181) sustained a fracture in the thoracic areas of the spine. Analysis of the medical records of the patients and the number of vertebral fractures showed that the largest number of respondents had sustained two (30%; *n* = 73), or a single fracture (28.4%; *n* = 69). In turn, every fifth woman (20.2%; *n* = 49) had sustained three fractures, and 13.6% four fractures (*n* = 33). The remainder 7.8% (*n* = 19) had sustained at least five fractures. On average, the women in the study had sustained 2.51 ± 1.49 fractures. Less than a half of respondents (42%; *n* = 102) were dissatisfied with their state of health, whereas 37.9% (*n* = 92) were neither satisfied nor dissatisfied. The replies indicating satisfaction with life were provided by only 16% of respondents (*n* = 39), while 4.1% of women (*n* = 10) were very dissatisfied with their state of health. The mean assessment of satisfaction with life was 2.66 ± 0.79, on a scale of 1–5.

Statistical analysis demonstrated that respondents were characterized by the lowest quality of life being in the domain of overall perception of health (69.77 ± 20.87), while the highest was in the domain of physical functioning (33.6 ± 21.92), reporting less problems with performing household activities and mobility (standing up, bending, kneeling, walking).. The result concerning the level of overall quality of life (42.79 ± 16.81) showed an average assessment of the quality of life in the examined group (Table 1).

The selected variables concerning respondents’ health were significantly related with their quality of life. Analysis showed that the T-score value obtained in densitometric examination of the lumbar spine region significantly correlated at a level of *p* < 0.05 with the quality of life measured using the QUALEFFO-41. Considering the reverse design of the QUALEFFO-41, the higher the T-score value obtained in densitometric examination of the lumbar region of the spine, the lower the results concerning physical functioning (*r* = −0.231; *p* < 0.001) and overall quality of life (*r* = −0.169; *p* = 0.008), as presented in Table 2.

Our study also confirmed that the quality of life of the respondents was correlated with the duration of treatment, perception of pain, and the frequency of its occurrence. The longer the treatment, the worse the quality of life in the domains of physical functioning, social functioning, overall perception of health, psychological functioning; and the lower the overall quality of life. In turn, the more frequent pain experienced in the spine and back region and the longer the duration of experiencing pain, the lower the level of the quality of life in the domains of pain, physical functioning, social functioning, overall perception of health, psychological functioning; and the lower the overall quality of life. The strongest correlation was observed between the frequency of experiencing pain in the region of the spine and back, and the quality of life in the domain of pain (rho = 0.522; *p* < 0.001). This correlation may be defined as moderately strong.

The relationship between the quality of life of women and the place of residence was also investigated. The respondents who sustained a fracture in the lumbar region of the spine were characterized by a significantly lower quality of life in the domain of social functioning, compared to respondents who did not sustain a fracture in the lumbar region of the spine (t = −3.141; *p* = 0.002). However, no statistically significant relationship was found between women who sustained a fracture in the thoracic region of the spine and quality of life: they did not differ statistically at a level of *p* < 0.05 from the aspect of the quality of life from women who did not sustain a fracture in this region of the spine.

The women in the study experienced 0–11 various complaints resulting from osteoporotic vertebral fractures—4.5, on average. The number of complaints related with past osteoporotic vertebral fractures was significant at a level of *p* < 0.01 which correlated with the quality of life measured using the QUALEFFO-41. These correlations were strong or moderate in the domain of physical functioning and overall quality of life. The bigger the number of complaints related with past osteoporotic vertebral fracture, the lower the level of overall quality of life in the domains of pain, physical functioning, social functioning, overall perception of health, and psychological functioning (Table 3).

The respondents’ self-reported quality of life appeared to be well related with their actual quality of life. The higher overall satisfaction with life, the higher the level of the quality of life in the domains of pain, physical functioning, social functioning, overall perception of health, and psychological functioning measured using the QUALEFFO-41 (Table 4).

The higher the respondents’ satisfaction with the outcomes of treatment, the higher the level of the quality of life in the domains of: pain (rho = −0.404; *p* < 0.001), physical functioning (rho = −0.326; *p* < 0.001), social functioning (rho = −0.315; *p* < 0.001), overall perception of health (rho = −0.439; *p* < 0.001), psychological functioning (rho = −0.307; *p* < 0.001), and overall quality of life (rho = −0.385; *p* < 0.001). The strongest correlation was observed between the degree of satisfaction with the outcomes of treatment, and the quality of life in the domain of overall perception of health.

The relationship between the applied methods of treatment and the quality of life were also analyzed. Neither the pharmacological method of treatment applied in nearly all women (treatment) nor the surgical treatment applied in only a few patients were considered. It was confirmed that respondents who were recommended to lie down and rest in bed were characterized at a level of *p* < 0.05 by a lower quality of life in the domains of social functioning and overall perception of health, compared to those who did not receive such methods of treatment (QUALEFFO-41) (Table 5).

Women who received treatment using a corset or orthopaedic belt were characterized by a significantly lower quality of life in the domains of pain, physical functioning, social functioning, overall perception of health, and overall quality of life, compared to those who did not receive such treatment (Table 6).

It was confirmed that respondents who sustained a fracture when falling from a standing position were characterized by a significantly lower overall quality of life concerning pain, and a lower quality of life concerning pain, compared to respondents who were not aware that they had sustained vertebral fracture. Moreover, respondents who sustained a fracture when falling from a standing position had a significantly lower level of quality of life in the domain of physical functioning than women in whom the fracture was caused by minor trauma while performing daily life activities (Table 7).

The presented analysis shows that there occurs a relationship between the respondents’ age and their quality of life. The older the age, the worse the quality of life in the domains of pain, physical functioning, social functioning, overall perception of pain, psychological functioning, and lower overall quality of life (Table 8).

Statistically significant differences were observed between women living in urban and rural areas, and their quality of life. Respondents living in rural areas had significantly lower quality of life in the domains of social functioning (t = −3.129; *p* = 0.002), overall perception of health (t = −3.501; *p* < 0.001), and psychological functioning (t = −2.173; *p* = 0.031), compared to urban inhabitants.

Analysis also confirmed that respondents who were married or were in a partnership relationship had a significantly higher quality of life in the domains of pain (t = −3.191; *p* = 0.002), physical functioning (t = −4.461; *p* < 0.001), social functioning (t = −2.737; *p* = 0.007), overall perception of health (t = −3.086; *p* = 0.002), psychological functioning (t = −4.414; *p* < 0.001), and overall quality of life (t = −4.572; *p* = 0.002), compared to those who were single (Table 9).

Analysis of variance showed significant differences between the level of education and the quality of life measured by means of QUALEFFO-41. In view of the ordinal character of the variable *education*, instead of correlation analysis, different testing was applied, because respondents with higher education were characterized by a significantly higher quality of life in all domains than among all the remainder. It turn, there were few statistically significant differences between the remaining groups. In the domains of pain and psychological functioning, respondents with higher education had a significantly higher quality of life compared to those with primary/junior high school, primary vocational, and secondary school education. In the domain of physical functioning, respondents with higher education had a significantly higher quality of life than those with primary/junior high school, primary vocational, and secondary school education, as did respondents who had post-secondary education compared to women with primary/junior high school education. In the domains of social functioning and overall perception of health, respondents with higher education were characterized by a significantly higher quality of life compared to those with primary/junior high school, primary vocational, secondary school, and post-secondary school education. A significantly higher quality of life was experienced by respondents with higher education compared to those with other types of education, and respondents with post-secondary education compared to those with primary/junior high school and primary vocational education (Table 10).

A linear correlation was also observed between material standard of living and quality of life. The higher the material standard of living, the higher the quality of life in the domains of pain (rho = −0.194; *p* = 0.002), physical functioning (rho = 0.346; *p* < 0.001), social functioning (rho = −0.418; *p* < 0.001), overall perception of health (rho = −0.327; *p* < 0.001), psychological functioning (rho = −0.310; *p* < 0.001), and overall quality of life (rho = −0.36; *p* < 0.001) (Table 11).

## 4. Discussion

The consequences of osteoporosis, a disease with a chronic course, concern many areas of functioning and the organization of life. They are not only the cause of physical dysfunctions, but also the lack of psychological comfort due to pain, suffering, or limitations, or even the loss of efficiency in carrying out daily activities [5]. Osteoporosis is characterized by a slow ‘quiet’ course, and frequently the occurrence of a low-energy fracture is the cause of diagnosis. Approximately 70% of fractures of the vertebral bodies in the course of osteoporosis are ‘clinically silent’ and remain undiagnosed [18]. Only every second case of osteoporotic vertebral fracture is the consequence of a fall and causes high intensity acute pain. Fractures usually occur while performing routine daily life activities, involving stumbling, standing up or sitting down, or even coughing or sneezing [26,27]. The consequence of compression fractures of the spine are most often chronic back pain, reduced mobility within the spine, and aggravation of thoracic kyphosis [28].

The occurrence of low-energy vertebral fracture causes an 11-fold increase in the risk of a subsequent fracture within the spine, and a 2–3-fold increase in the risk of fractures in other major locations [16]. Unfortunately, only approximately 10–20% of people receive pharmacological therapy after the occurrence of the first osteoporotic fracture [29].

Fractures of vertebral bodies and their consequences in the form of noxious pain complaints, deformation of the silhouette and decreased efficiency in daily activities, result in depressive disorders, loss of independence, social isolation, problems with adaptation to new health circumstances, decrease in the quality of life, or even death [26].

Therefore, the assessment of the quality of life of patients who have undergone vertebral fracture should evoke great interest among researchers, especially considering the long-term, progressive course of the disease, and the fact that the complaints may, to a great extent, limit the functioning of the patient.

In Poland, few studies have been conducted to-date concerning the quality of life of patients after osteoporotic vertebral fracture, and even fewer attempt to establish, as we have, what factors are associated with the patients’ quality of life, hypothetically causing its increase of deterioration.

The majority of studies carried out in Poland and worldwide focus on determining whether the quality of life of patients with osteoporosis (assumed to be lower) differs from the quality of life of the healthy. However, it should be remembered that establishing the factors that determine the level of the quality of life specifically among the ill may allow the undertaking of actions aimed at improving the quality of life of patients.

As previously mentioned, in the relevant literature, studies may be encountered aimed at comparing the quality of life of patients with osteoporosis and the healthy. A study by Bączyk et al. [30], which included a group of 304 women aged 50–69 treated in the Outpatient Department for Menopause and Osteoporosis at the Maternity and Gynaecological Hospital of the Medical University in Poznań, aimed to assess the quality of life of women with and without vertebral fractures. Analysis of results showed that the level of the quality of life of women with osteoporosis and osteopenia was higher than an overall level of quality of life of women after vertebral fractures in the study.

Moreover, women who participated in our study had worse quality of life compared to the mean QUALEFFO-41 results presented by either Stanghelle et al. (26.7 ± 13.1) who examined women in Norway, or Singh et al. (30.1 ± 3.82) on women in India [31,32]. In turn, the results of QUALEFFO-41 obtained in the present study were similar to those obtained in a study by Ciubean et al. among Romanian women (44.48 ± 42.34) [33]. While referring to the results obtained in this study, it is worth mentioning that even lower results of the quality of life (M = 49.38) were obtained by Drozd et al. [33], who arrived at slightly different conclusions in a study conducted in two Outpatient Departments for the Treatment of Osteoporosis in Lublin. The study covered a group of post-menopausal women aged 54–86, who received ambulatory treatment due to osteoporosis. The quality of life was also assessed using the QUALEF-FO-41. Unlike our research, apart from lower overall quality of life, the examined women showed a lower quality of life in the domain of physical functioning (M = 39.41), overall perception of health (M = 79.55), and assessment of pain (M = 53.45).

The study carried out by Drozd et al. [33] covered a group of only 55 women with osteoporosis; therefore the current study, due to the size of the sample, may be considered as more representative.

Despite the fact that fractures decrease the quality of life, they do not affect all its components to the same degree. In the present study, the respondents were characterized by the lowest quality of life being in the domain of general perception of the state of their health, whereas the highest was in the domain of physical functioning (household chores). The possible reason for which few respondents reported difficulties with cooking, washing dishes and shopping was that they obtained help from people close to them. It is worth mentioning that the respondents’ overall quality of life was not low, but mediocre. This may be due to their relatively high evaluation of physical functioning. Meta-analysis carried out by Al-Sari et al. demonstrated that a lower quality of life of persons with osteoporosis is manifested more by physical than psychological functioning [34]. This relationship was also confirmed by a cross-sectional study including 893 Australian men aged 24–92 [35]. A study conducted by Vujasinocić et al. who also used the QUALEF-FO-41 demonstrated that women with osteoporosis showed poor functioning in every domain of the scale [36].

Osteoporotic fractures cause chronic pain, the consequence of which is decreased general evaluation of health. In our study the respondents were characterized by the lowest quality of life in the domain of an overall perception of their state of health and mediocre evaluation in the domain of pain. Leidig-Bruckner et al. and Ostrowska et al. found that persons who perceive pain are inclined to report a lower assessment of the quality of their own life [37,38]. Pain caused by vertebral fracture is one of the main factors affecting the quality of life, because it deteriorates the quality of sleep, mobility, and generally the emotional health of patients [31]. A study conducted in the surgical clinic of the Instituto Italiano Auxologico during a period of approximately 4 months, among 100 women with osteoporosis with or without fractures aged 50–85, demonstrated that pain was present in 50% of cases, and in 26% of cases for a period longer than 4 months [39]. According to a study carried out in Italy assessing an overall perception of health of women with osteoporosis, more than a half of them reported general malaise. When comparing their present level of state of health with that from 10 years ago, 58% of women aged under 65—and 83% of those aged 65 and over—indicated a deterioration in perception of health and overall quality of life [39].

Many studies show that with age, disorders of bone homeostasis and loss of trabecular bone occur which additionally increase the risk of fractures. Our study demonstrated that age is statistically significantly related with the quality of life of women who had undergone fractures. The overall quality of respondents’ life deteriorates with age, as well as the quality of life in each investigated domain of the quality of life. Our observations are in accordance with the results obtained by Singh et al., Özsoy-Ünübol et al., Drozd et al., and Ramirez-Perez et al. [33,40,41,42]. A decrease in the quality of life with age may result from a longer life with illness and progressive disability enhanced by fractures.

A cross-sectional study conducted in 55 centres in Italy engaged in the diagnostics of osteoporosis, which covered 885 women at post-menopausal age who had radiographic examination of the spine performed, showed that the level of the quality of life measured by means of the QUALEFFO-41 questionnaire depends on the grade of deformation and location of the fracture. The occurrence of a fracture in the lumbar region was associated with a lower quality of life, compared to women in whom the fracture concerned the thoracic spine. According to the researchers, the grade of deformation may exert a greater effect on the level of the quality of life than the location of the fracture, which results from the fact that the ratio between moderate-severe and mild fractures is clearly highest in the lumbar region of the spine [43]. The current study demonstrated that women who sustained a fracture in the lumbar spine region had a significantly lower quality of life in the domain of social functioning (QUALEFFO-41) compared to those who did not sustain fractures in this region.

In a study by Abimanyi-Ochom et al. conducted with the use of the EQ-5D questionnaire, the researchers observed a mean decrease in the quality of life for all locations of fractures, with the greatest decrease noted in the case of fracture of the proximal spine. In the Swedish study using the same protocol, Ström et al. obtained similar results [44,45]. In turn, a study carried out in Austria by Jahelka et al. [46] which included a sample of 222 patients with osteopenia and osteoporosis treated in the geriatric rehabilitation ward showed a significantly lower quality of life for patients who had undergone fracture, irrespective of its location.

Similar to our study, a further study conducted in Mexico by Ramirez-Perez et al. [42] demonstrated that the aspects exerting the most important effect on the quality of life of patients were marital status and level of education, as well as the place of residence. Respondents who lived in rural areas had a significantly lower quality of life in the domains of social functioning, overall perception of health, and psychological functioning, compared to urban inhabitants. The respondents who were married or remained in a partnership relationship, as well as those with higher education, were characterized by a higher quality of life in all domains of QUALEFFO-41, compared to those who were single and without higher education. Such results most probably result from the fact that having a life partner relatively often allows for better support in illness, and better education is associated with greater skills in seeking assistance and better knowledge about ways of coping in illness. In turn, a lower quality of life of rural inhabitants may be related with worse possibilities for coping in the situation of illness, e.g., more difficult access to health facilities, lack of specialists in rural outpatient units, thus hindered access to physicians, rehabilitation centres and pharmacies.

The results of the study presented here demonstrate that the lower the material standard of living, the lower the quality of life in all domains of QUALEFFO-4, and the lower level of overall quality of life.

Our results are not innovative in this regard, and were consistent with those obtained by, e.g., Bączyk et al. [47], nor are they surprising, since a higher material standard of living is associated with the greatest possibility of rapid contact with visits to private physicians, of purchasing rehabilitation equipment, of access to private rehabilitation, and also greater possibilities of adjusting lodging to the needs of the ill person.

The present study showed that the consequences of osteoporosis and vertebral fractures in the form of pain complaints, progressing deformation of the silhouette, and limitation of physical fitness are the cause of considerable deterioration in the quality of life of women.

*Study Limitation.* Our study has some limitations that should be addressed. In this study, important characteristics were applied; however, confounding factors cannot be totally excluded, which could remain unmeasured. It is probable that not all actions were described that aimed at the elimination of the potential sources of bias in our study. One limitation of our study is that it included a selected group of women from health institutions in Lublin. The study did not cover all facilities in Poland, and so may not reflect the results for the whole of Poland. Undoubtedly, the limitation of the study is a small number of participants. It would be justifiable to compare the results of this study with those of a larger group of women from various centres with a control group. The above-mentioned disadvantages create an opportunity for further research.

## 5. Conclusions

The level of the quality of life of women who had undergone osteoporotic vertebral fractures was mediocre. While reviewing literature, no reports were found concerning the problem addressed in the present study. To the best of the authors’ knowledge, in Poland there are no studies presenting such an analysis.

The results of our study suggest that the ill women require a multidisciplinary, individual approach in order to make a quick and correct diagnosis of the relevant deficits in physical, social, and psychological spheres. The presented results may be used for the development of new prophylactic strategies aimed at improvement in the quality of life of women with osteoporotic vertebral fractures. Education would be advisable concerning the prevention and effective elimination of complaints related with the illness, which would help these women to function more comfortably in society.

In order to recognize problems resulting from osteoporotic fractures and improve the monitoring of the effectiveness of treatment, in the future it is planned to expand the study by including a larger number of women, increasing its territorial scope, and by a comparison with women not burdened with fractures.

## Figures and Tables

**Table 1:** Descriptive statistics of the quality of life measured using QUALEFFO-41.

QUALEFFO-41 Domains	Min.	Max.	M	Me	Mo	SD
Pain	0.00	90.00	48.64	50.00	55.00	19.65
Physical functioning	2.94	95.59	33.60	29.41	4.41	21.92
Social functioning	25.00	100.00	62.37	66.25	66.00	18.07
Overall perception of health	25.00	100.00	69.77	75.00	83.00	20.87
Psychological functioning	8.00	81.00	43.14	44.00	47.00	15.95
**Overall quality of life**	8.00	85.00	42.79	43.00	44.00	16.81

**Table 2:** Correlations between the quality of life measured using QUALEFFO-41 and T-score.

QUALEFFO-41 Domains	T-Score from Densitometry of the Lumbar Spine Region	
	R	*p*
Pain	−0.111	0.085
Physical functioning	−0.231	0.000 **
Social functioning	−0.012	0.854
Overall perception of health	−0.115	0.073
Psychological functioning	−0.067	0.302
**Overall quality of life**	−0.169	0.008 **

Statistically significant ** *p* < 0.01.

**Table 3:** Quality of life measured using QUALEFFO-41 and the number of complaints related with past osteoporotic vertebral fracture.

QUALEFFO-41 Domains	Number of Symptoms Related with Osteoporosis and/or Past Osteoporotic Vertebral Fracture	
	R	*p*
Pain	0.591	0.000 **
Physical functioning	0.685	0.000 **
Social functioning	0.449	0.000 **
Overall perception of health	0.569	0.000 **
Psychological functioning	0.586	0.000 **
**Overall quality of life**	0.705	0.000 **

Statistically significant ** *p* < 0.01.

**Table 4:** Correlations between the quality of life measured using the QUALEFFO-41 and self-reported state of health.

QUALEFFO-41 Domains	Self-Reported State of Health	
	Rho	*p*
Pain	−0.552	0.000 **
Physical functioning	−0.607	0.000 **
Social functioning	−0.519	0.000 **
Overall perception of health	−0.751	0.000 **
Psychological functioning	−0.577	0.000 **
**Overall quality of life**	−0.658	0.000 **

Statistically significant ** *p* < 0.01.

**Table 5:** Quality of life measured using the QUALEFFO-41 and treatment by the method of lying down and resting in bed.

QUALEFFO-41 Domains	Lying down and Resting in Bed				T-Student Test	
	No		Yes			
	M	SD	M	SD	T	*p*
Pain	48.45	21.44	48.74	18.71	−0.109	0.913
Physical functioning	31.71	21.84	34.60	21.96	−0.979	0.328
Social functioning	58.74	17.59	64.29	18.07	−2.301	0.022 *
Overall perception of health	66.06	20.23	71.72	20.99	−2.025	0.044 *
Psychological functioning	43.75	14.62	42.81	16.65	0.436	0.664
**Overall quality of life**	41.39	16.10	43.53	17.18	−0.941	0.347

Statistically significant * *p* < 0.05.

**Table 6:** Quality of life measured using the QUALEFFO-41 and treatment using a corset or orthopaedic belt.

QUALEFFO-41 Domains	Using a Corset or Orthopaedic Belt				T-Student Test	
	No		Yes			
	M	SD	M	SD	T	*p*
Pain	47.25	19.51	54.78	19.30	−2.342	0.020 *
Physical functioning	31.96	21.41	40.82	22.89	−2.473	0.014 *
Social functioning	61.27	18.06	67.24	17.48	−2.015	0.045 *
Overall perception of health	68.11	20.74	77.04	20.07	−2.624	0.009 **
Psychological functioning	42.86	16.22	44.33	14.85	−0.557	0.578
**Overall quality of life**	41.59	16.48	48.09	17,42	−2.364	0.019 *

Statistically significant * *p* < 0.05, ** *p* < 0.01.

**Table 7:** Quality of life measured using the QUALEFFO-41 and circumstances of sustaining vertebral fracture.

QUALEFFO-41 Domains	Do You Associate the Fact of Sustaining Osteoporotic Vertebral Fracture with:								
	Falling from A Standing Position (1)		Minor Trauma Caused by Daily Life Activities (2)		I Was not Aware of Vertebral Fracture (3)		ANOVA		
	M	SD	M	SD	M	SD	F	*p*	R.I.
Pain	56.55	17.73	49.83	19.45	46.02	19.78	3.717	0.026 *	1/3
Physical functioning	42.95	21.93	31.54	20.76	32.87	22.31	3.148	0.045 *	1/2
Social functioning	69.00	13.40	62.86	19.54	60.52	17.68	2.685	0.070	ns
Overall perception of health	75.90	15.41	68.48	21.43	69.24	21.44	1.461	0.234	ns
Psychological functioning	48.66	12.05	42.63	17.05	42.22	15.81	2.005	0.137	ns
**Overall quality of life**	50.28	13.98	41.94	17.18	41.66	16.83	3.335	0.037 *	1/3

Statistically significant * *p* < 0.05. Abbreviations: Min, minimum; Max, maximum; M, mean; Me, median; SD, standard deviation; ns, non-significan; R.I.—test difference.

**Table 8:** Correlation between the quality of life measured using the QUALEFFO-41 and age.

QUALEF-FO-41 Domains	Age (Years)	
	R	*p*
Pain	0.316	0.000 **
Physical functioning	0.518	0.000 **
Social functioning	0.448	0.000 **
Overall perception of health	0.385	0.000 **
Psychological functioning	0.315	0.000 **
**Overall quality of life**	0.497	0.000 **

Statistically significant ** *p* < 0.01.

**Table 9:** Quality of life measured using the QUALEFFO-41 and marital status and place of residence.

**QUALEFFO-41 Domains**	**Marital Status**				**Student’s *t*-Test**	
	**Married/In a Partnership Relationship**		**No Husband/Partner**			
	**M**	**SD**	**M**	**SD**	**T**	***p***
Pain	45.28	19.16	53.28	19.47	−3.191	0.002 **
Physical functioning	28.46	18.50	40.70	24.27	–4.461	0.000 **
Social functioning	59.71	19.34	66.05	15.49	−2.737	0.007 **
Overall perception of health	66.31	22.06	74.54	18.14	−3.086	0.002 **
Psychological functioning	39.43	15.86	48.25	14.69	–4.414	0.000 **
**Overall quality of life**	38.76	15.96	48.36	16.43	–4.572	0.000 **
	**Place of Residence**				**Student’s *t*-Test**	
	**Urban**		**Rural**			
	**M**	**SD**	**M**	**SD**	**T**	***p***
Pain	47.50	20.63	50.19	18.22	−1.056	0.292
Physical functioning	33.11	23.30	34.27	19.98	−0.406	0.685
Social functioning	59.32	19.15	66.52	15.63	−3.129	0.002 **
Overall perception of health	65.84	20.52	75.11	20.23	−3.501	0.001 **
Psychological functioning	41.24	16.43	45.71	14.97	−2.173	0.031 *
**Overall quality of life**	41.31	17.87	44.80	15.11	−1.601	0.111

Statistically significant * *p* < 0.05, ** *p* < 0.01.

**Table 10:** Quality of life measured using the QUALEFFO-41 and the level of education.

Domains QUALEFFO-41	Education										ANOVA		
	Primary/Junior High School (1)		Primary Vocational (2)		Secondary School (3)		Post-Secondary School (4)		Higher (5)				
	M	SD	M	SD	M	SD	M	SD	M	SD	F	*p*	R.I.
Pain	51.62	22.42	54.40	18.19	49.24	17.68	43.75	17.51	34.19	18.62	7.116	0.000 **	1/5 2/5 3/5
Physical functioning	43.08	26.90	39.92	19.59	32.35	21.21	27.68	14.88	15.23	14.10	10.819	0.000 **	1/5 2/5 3/5 1/4
Social functioning	71.13	11.71	68.14	14.81	63.26	14.41	62.84	16.81	35.47	15.92	32.387	0.000 **	1/5 2/5 3/5 4/5
Overall perception of health	76.11	21.22	75.76	17.93	70.06	19.13	67.04	19.96	49.48	19.58	11.567	0.000 **	1/5 2/5 3/5 4/5
Psychologicalfunctioning	49.00	17.70	45.20	13.38	45.61	13.48	39.29	13.79	28.87	18.50	9.862	0.000 **	1/5 2/5 3/5
**Overall quality of life**	50.14	19.23	47.84	14.11	43.03	14.75	38.61	14.11	25.03	13.35	15.560	0.000 **	1/5 2/5 3/5 4/5 1/4 2/4

Statistically significant ** *p* < 0.01.

**Table 11:** Correlation between quality of life measured using the QUALEFFO-41 and material standard of living.

QUALEFFO-41 Domains	Material Standard	
	Rho	*p*
Pain	−0.194	0.002 **
Physical functioning	−0.346	0.000 **
Social functioning	−0.418	0.000 **
Overall perception of health	−0.327	0.000 **
Psychological functioning	−0.310	0.000 **
**Overall quality of life**	−0.366	0.000 **

Statistically significant ** *p* < 0.01.

## Data Availability

The data presented in this study are available on request from the corresponding author. The data are not publicly available due to privacy restrictions.

## Supplementary Materials

The following supporting information can be downloaded at: https://www.mdpi.com/article/10.3390/ijerph191912237/s1, File S1: Research Questionnaire.
