# Supplementary material for: Assessment of the Quality of Life of Women after Osteoporotic Vertebral Fracture with Consideration of Socio-Demographic Characteristics and Selected Factors Concerning the State of Health
Source: Int J Environ Res Public Health. 2022 Sep 27;19(19):12237. doi: 10.3390/ijerph191912237 (PMC9566652; doi:10.3390/ijerph191912237)
Supplement: Supplementary file 1 [file ijerph-19-12237-s001.zip › ijerph-1890101-supplementary.pdf]

Research Questionnaire

ACCEPTANCE OF ILLNESS AND QUALITY OF LIFE OF WOMEN AFTER OSTEOPOROTIC VERTEBRAL FRACTURE

Dear Madam,

I kindly request your assistance in pursuing this research project by completing the questionnaire. The study is ***anonymous*** *and will be used exclusively for scientific purposes.*

In each question please mark only one answer, unless otherwise requested. Please provide honest and truthful answers.

Please mark with an ***X*** *your selected answers, or complete the places marked with dots.*

1. **Age** …………years
2. **Body weight**............ kg
3. **Height** ……….. cm
4. **Marital status**
   a) Never married
   b) Married/in a partnership relation
   c) Divorced
   d) Widowed
5. **Place of residence**
   a) Urban
   b) Rural
6. **Education**
   a) Primary/junior high school
   b) Vocational
   c) Secondary school
   d) Post-secondary school
   e) Higher
7. **Material standard**

a) Very good
b) Good
c) Sufficient
d) Poor
e) Very poor

8. **Current occupational status**
   a) Intellectual worker
   b) Physical worker
   c) Unemployed
   d) Retired/ health allowance
   e) Other *(what?)*……………

9. **How much time has elapsed since the last menstruation?**.........................
10. **Do you suffer from any of the below-mentioned diseases?***(you can mark more than one answer)*
    a) Type I diabetes I (requiring subcutaneous injection of insulin)
    b) Congenital bone fragility
    c) Hyperthyroidism untreated for a long time
    d) Hypogonadism (primary ovarian insufficiency), or premature menopause (climacterium) before the age 45
    e) Chronic malnutrition or malabsorption syndrome
    f) Chronic liver disease
    g) Rheumatoid arthritis RA (rheumatism)
    h) Other chronic diseases *(what?)*…………………
11. **Has fracture of the proximal end of the femur (hip) occurred in your father or mother?**
    a) Yes
    b) No
12. **Have you had fracture risk assessed using the Fracture Risk Assessment Tool - FRAX calculator?**
    a) Yes, the risk of bone fracture was low
    b) Yes, the risk of bone fracture was on a mediocre level
    c) Yes, the risk of bone fracture was high
    d) Yes, but I do not remember the result of the examination
    e) No
    f) I have never heard about such an examination
13. **Which part of the skeleton did your bone density examination (densitometry) concern?** *(you may mark more than one answer)*
    a) Proximal end of the femur (hip)
    b) Lumbar spine
    c) Radial bone (in the wrist area)
    d) Calcaneus
14. **What other examinations have you undergone in order to confirm osteoporosis and/or vertebral fracture?** *(you may mark more than one answer)*
    a) Radiological examination of the spine RTG
    b) Computed tomography
    c) Magnetic resonance
    d) Morphometric densitometry (vertebral fracture assessment – VFA)

e) Laboratory tests of blood and urine
f) Other examinations *(what?)…………………..*
g) None of the above-mentioned

15. **How long have you been treated for osteoporosis?**
    a) Less than 1 year
    b) From 1 - 5 years
    c) More than 5 - 10 years
    d) More than 10 years
16. **Do you experience pain in the region of the spine and back?**
    a) Yes, all the time
    b) Yes, frequently
    c) Yes, sometimes
    d) Yes, rarely
    e) No, never

NOTE: in the CASE OF CHOOSING ANSWER **e)** PLEASE GO TO QUESTION **18**

17. **Since when have you experienced pain the region of the spine and back?**
    a) Less than 1 year
    b) From 1 - 5 years
    c) More than 5 - 10 years
    d) More than 10 years
18. **Do you associate the fact of osteoporotic vertebral fracture with:**
    a) Falling from a standing position
    b) Minor injury caused by activities of daily living, e.g. getting out of bed, bending down, lifting weights, tripping over obstacle, sudden sneezing, cough, driving without shock-absorption, etc.
    c) I was unaware that I had vertebral fracture, because there was no perceptible injury, and I learned about this fact during a visit to a doctor
19. **Do you have knowledge concerning the number of past osteoporotic vertebral fractures?**
    a) Yes (how many?) …………
    b) No
20. **Apart from vertebral fractures have you sustained other osteoporotic bone fractures?** *(you may mark more than one answer, please provide the number of past fractures next to the selected answer)*
    a) Yes, fracture of the proximal end of the femur (hip)...........
    b) Yes, fractures of the distal end of the radius (wrist)…….
    c) Yes, fractures other than the above-mentioned typical of osteoporosis (shoulder bone, collarbone, shoulder blade, femur, shin bone, pelvis, ribs) ………
    d) No
21. **What symptoms have you observed in association with osteoporosis and/or past osteoporotic vertebral fracture?** *(you can mark more than one answer)*
    a) Acute pain (sometimes girdle pain accompanied by tension of adjacent muscles) in the middle-lower region of the thoracic spine or lumbar-thoracic spine
    b) Chronic back pain at rest and/or while performing activities of daily living
    c) Intensification of pain while sneezing, coughing and defecation
    d) Difficulty with sitting down or standing up
    e) Sensory disturbances and lower limb paresis

f) Reduction in a normal range of motion in the joints
g) Problems with mobility
h) Curvature of the spine, slouching ('widow's hump')
i) Reduction in height
j) Decline in circulatory and respiratory efficiency
k) Intestinal disorders
l) Deterioration of mood, depression
m) Other symptoms *(what?)*……………………………
n) I do not experience any symptoms

22. What methods of treatment have been applied in association with the fact of your osteoporotic vertebral fracture?*(you can mark more than one answer)*

a) Pharmacological treatment (taking medicines recommended in treatment of osteoporosis)
b) Lying down and resting in bed
c) Use of a corset or orthopaedic belt
d) Isometric exercises
e) Rehabilitation procedures, e.g. physical therapy, hydrotherapy
f) Surgical treatment of vertebral fracture (balloon kyphoplasty or vertebroplasty)
g) Other *(what?)*…………………………..

NOTE: in the CASE OF CHOOSING ANSWER **h)** PLEASE GO TO QUESTION **24**

23. **To what extent are you satisfied with the outcomes of the applied treatment?**

a) Very satisfied
b) Satisfied
c) Neither satisfied nor dissatisfied
d) Dissatisfied
e) Very dissatisfied

24. What behaviours concerning life style do you present?

a)	Do you consume milk and dairy products every day?	Yes	No
b)	Do you apply diet rich in fish, vegetables and fruits?	Yes	No
c)	Do you try to apply diet with an adequate amount of proteins (meat, eggs, dairy products, nuts, cereals, legumes)?	Yes	No
d)	Do you take preparations containing calcium and vitamin D?	Yes	No
e)	Do you limit the consumption of products rich in phosphorus, such as meat, cold cuts, yellow cheeses?	Yes	No
f)	Do you try to reduce the consumption of salt, sugar, and preservatives?	Yes	No
g)	Do you apply a low-calorie diet or intensively lose weight?	Yes	No
h)	Do you reasonably use solar radiation?	Yes	No
i)	Are you currently smoking cigarettes?	Yes	No
j)	Do you consume 3 or more units of alcohol daily? (1 unit is a shot-glass of vodka, glass of wine, mug of beer)	Yes	No
k)	Do you regularly drink strong coffee or tea in an amount of more than 2–3 cups daily?	Yes	No
l)	Do you maintain everyday physical activity, e.g. walks, nordic walking (march with poles), dancing, riding a bicycle, aerobic, stretching exercises, yoga, swimming, etc.?	Yes	No

ł)	Do you try to prevent falls (removing protruding door thresholds at home, slippery rugs, installation of handrail in the bathroom, good lighting, wearing stable footwear on not slippery sole, etc.)?	Yes	No
m)	Do you apply or did you apply hormone replacement therapy HRT and/or herbal remedies alleviating the symptoms of menopause?	Yes	No
n)	Do you currently take orally glucocorticosteroids or have you taken them for longer than three months at a dose equivalent to 5 mg prednisolone?	Yes	No
o)	Have you been taking for a long time any of the following drugs: blood-thinning medicines, antiepileptic drugs, sedatives, diuretics, thyroid hormones?	Yes	No
p)	Do you follow doctor's recommendations and regularly take the prescribed drugs?	Yes	No
r)	If necessary, do you use advice and follow the recommendations of a medical specialist, e.g. cardiologist, neurologist, and ophthalmologist?	Yes	No

25. **To what extent osteoporosis and/or vertebral fracture exerted an effect on the deterioration of your quality of life?**

a) To a very great extent
b) To a great extent
c) Neither to a great nor small extent
d) To a small extent
e) Had no effect

Thank you very much for completing the questionnaire and the time devoted. Please continue to the next questionnaires.

Yours faithfully,
